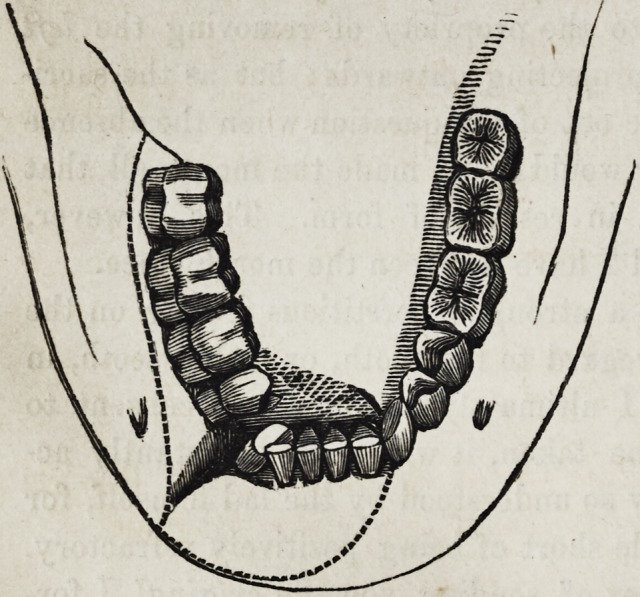# Case of Compound Fracture of Lower Jaw

**Published:** 1853-10

**Authors:** Charles Brown

**Affiliations:** Surgeon Dentist, Brighton, England.


### ARTICLE VI.

### *Case of Compound Fracture of Lower Jaw.* By Charles Brown, Surgeon Dentist, Brighton, England.

Lieut. V., royal artillery, was received into the Royal Ordinance Hospital with compound fracture of the inferior maxillary bone, caused by a kick from a horse in the riding school. I saw him the day after the injury, but could make no satisfactory examination, by reason of the tumified state of the parts, and the extravasation of blood into the sublingual tissues. I enjoined strict quiet, and exhibited a cathartic mixture. On the third day the tumefaction had sufficiently subsided to allow of an examination.

The blow appeared to have been given rather anterior to the external foramen on the right side, fracturing the bone completely across, (see diagram,) and taking a right and left oblique direction, upwards from the base of the fracture, and splitting away the inner alveolar plate and teeth as far as the dens sapientiæ on the right side, and on the left the alveolar plate posterior to the incisor and including the processus innomenatus; (see dotted lines,) thus dividing the bone into three distinct fragments.

A very shallow tin, charged with wax, was with difficulty passed over the teeth of the comparatively uninjured side of the jaw, and a slight impression obtained of their crowns, by the aid of which a model was executed sufficiently correct to allow of a metal capping being struck up in the usual way, and armed with a strong bar to bear against and support the detached alveolar plate and teeth. At our next visit reduction of the fractured portions was effected. The capping, with its bar, was adjusted in its place; a strong double tailed gummed bandage supported the jaw externally. Antiphlogistic treatment was adopted, and the case progressed to a satisfactory issue in little more than four weeks; a slight pucker upon the chin, where the blow took effect, and the loss of one tooth being the only remaining indication of the injury.

## Figures and Tables

**Figure f1:**